# Interaction of Vitamin D Receptor with HLA DRB1*0301 in Type 1 Diabetes Patients from North India

**DOI:** 10.1371/journal.pone.0008023

**Published:** 2009-12-02

**Authors:** Neetu Israni, Ravinder Goswami, Avinash Kumar, Rajni Rani

**Affiliations:** 1 Molecular Immunogenetics Group, National Institute of Immunology, New Delhi, India; 2 Endocrinology and Metabolism Department, All India Institute of Medical Sciences, New Delhi, India; Ohio State University Medical Center, United States of America

## Abstract

**Background:**

Type 1 diabetes (T1D) is a multifactorial autoimmune disorder where interaction and integration of immune response genes along with environmental factors play a role in autoimmune destruction of the insulin producing Pancreatic Beta cells.

**Methodology/Principal Findings:**

We have studied four single nucleotide polymorphisms (*FokI* site in Exon 2, *BsmI* and *ApaI* sites in Intron 8 and *TaqI* site in exon 9) in the *vitamin D receptor* (*VDR*) gene using PCR-RFLP and *HLA-DRB1* alleles using PCR and hybridization with sequence specific oligonucleotide probes and studied their interaction using LD based statistics for non-linked loci followed by sequence analysis of the vitamin D response element (VDRE) present in the promoter region of HLA-DRB1*0301. Haplotypes, constructed using SHEsis program for four single nucleotide polymorphisms in the *VDR* gene, were studied for their interaction with *HLA-DRB1* alleles in 233 T1D patients and 191 healthy controls from North India. A significant increase of haplotypes *FBAt* and *fBAT* (p<0.02, OR = 1.44 and p<0.002, OR = 3.23 respectively) was observed in the patients. Both the haplotypes *FBAt* and *fBAT* were significantly increased in male patients with age at onset less than 18 years; however, *fBAT* was significantly increased in female patients irrespective of their age at onset. LD based statistics showed significant interaction between the high producer *F* and *T* alleles with HLA-DRB1*0301. *F* and *T* alleles of *VDR* have been shown to contribute to *VDR* mRNA independently. The promoter sequence analysis of HLA-DRB1*0301 showed presence of VDRE involved in higher expression of HLA-DRB1*030, which was confirmed by flow cytometry and real time PCR analysis.

**Conclusions/Significance:**

These data suggest that the interaction between *VDR* and *HLA* alleles is mediated by VDRE present in the promoter region of HLA-DRB1*0301 allele, which may be detrimental for the manifestation of T1D in the absence of 1,25-(OH)_2_D_3_ in early childhood due to poor expression of DRB1*0301 in the thymus resulting in autoimmunity.

## Introduction

Type 1 diabetes (T1D) is a multifactorial, autoimmune disorder where the insulin producing pancreatic beta cells are destroyed by one's own immune system. The disorder occurs with an incidence of 10.5/100,000/year in India [1]. T1D develops as a result of complex interaction of many genetic and environmental factors leading to autoimmune destruction of the insulin producing Pancreatic Beta cells. While 20 genomic intervals have been implicated for the manifestation of the disease, role of an intricate network of the products of these genes cannot be ruled out. We have shown earlier that simultaneous presence of DRB1*0301 along with homozygous *INS-VNTR* class-I was significantly increased (p<10^−8^) in T1D patients, giving a relative risk of 70.81 [2]. Simultaneous presence of high secretor genotypes of *TNF-α -308 (GA + AA*) along with high secretor genotypes of *IL-6, IL-10* and *TGF-β1* were also significantly increased in T1D patients. Low secretor genotype of *TNF- α -308 GG* along with low secretor genotypes of *IFN-γ*, high secretor genotypes of *IL-6*, and *TGF- β1* were protective. This effect of *TNF-α* high secretor genotype was independent of predisposing HLA-DRB1*0301 [3]. To further understand the intricate network of genes regulating the immune responses, we have studied the interaction of *vitamin D receptor (VDR)* polymorphic alleles with predisposing *HLA* alleles in T1D patients using Linkage Disequilibrium (LD) based statistics between two unlinked loci.

Vitamin D Receptor (VDR) is a ligand dependent transcription factor that belongs to the super family of the Nuclear Hormone Receptors [4]. The ligand for VDR is Vitamin D3 i.e., 1,25-(OH)_2_D_3_ which mediates its biological actions through VDR. Binding of 1,25-(OH)_2_D_3_ induces conformational changes in VDR which promotes its hetero-dimerization with Retinoid X Receptor (RXR), followed by translocation of this complex into the nucleus. The RXR-VDR heterodimer binds to the vitamin D_3_ responsive elements (VDRE) in promoter regions of 1,25-(OH)_2_D_3_ responsive genes[5], which in turn results in the regulatory function of 1,25-(OH)_2_D_3_. In the absence of classical responsive elements, 1,25-(OH)_2_D_3_ may control the expression of some genes like cytokine genes by targeting inducible transcription factors like NFAT in IL-2 in a sequence specific manner [6]. 1,25-(OH)_2_D_3_ has been shown to have an important immuno-modulatory role since it represses transcription of *IL-2* [7], [8], *IFN-γ* [9], *GM-CSF* [10] and *IL-12* [11] and up regulates the production of Th2 cytokines IL-4 and TGF-β1 [12], thereby inhibiting the overall Th1 responses. It has been shown to enhance the development of TH2 cells via a direct effect on naive CD-4 cells [5]. Besides, 1,25-(OH)_2_D_3_ has also been shown to modulate the expression of *HLA* class-II alleles on monocytes and human bone cells [13], [14]

In NOD mice, administration of 1,25-(OH)_2_D_3_ before the onset of Insulitis, has been effectively shown to prevent the disease progression. However, this treatment was found to be ineffective when Insulitis had already been established. Treatment of adult NOD mice with 1,25-(OH)_2_D_3_ analog has also been shown to be effective [15]–[18]. Similarly, in humans, vitamin D supplementation in early childhood has been shown to reduce the incidence of T1D [19], [20]. Since 1,25-(OH)_2_D_3_ is a VDR ligand, we have studied the *VDR* gene polymorphisms and their interaction with the most predisposing *MHC* alleles to investigate their role, if any, in the pathophysiology of T1D. The VDR single nucleotide polymorphisms (SNPs) studied include the T>C SNP in exon2 initiation codon detected with *FokI* restriction enzyme [21], the A>G SNP detected with *BsmI* [22] and G>T SNP detected with *ApaI* [23] located in Intron 8, and a silent C>T SNP [24] detected with *TaqI*, located in Exon 9. We studied the interaction between *VDR* alleles and predisposing *HLA* alleles using LD based statistics [25] and subsequently sequenced the promoter region of the predisposing *MHC* allele to detect the VDRE sequence which has been shown to modulate the expression of the HLA alleles [26], suggesting the functional implications of the statistically significant interaction.

## Results

### VDR *FokI, BsmI, ApaI* and *TaqI* Genotypes and Haplotypes in T1D Patients

**Figure 1:**
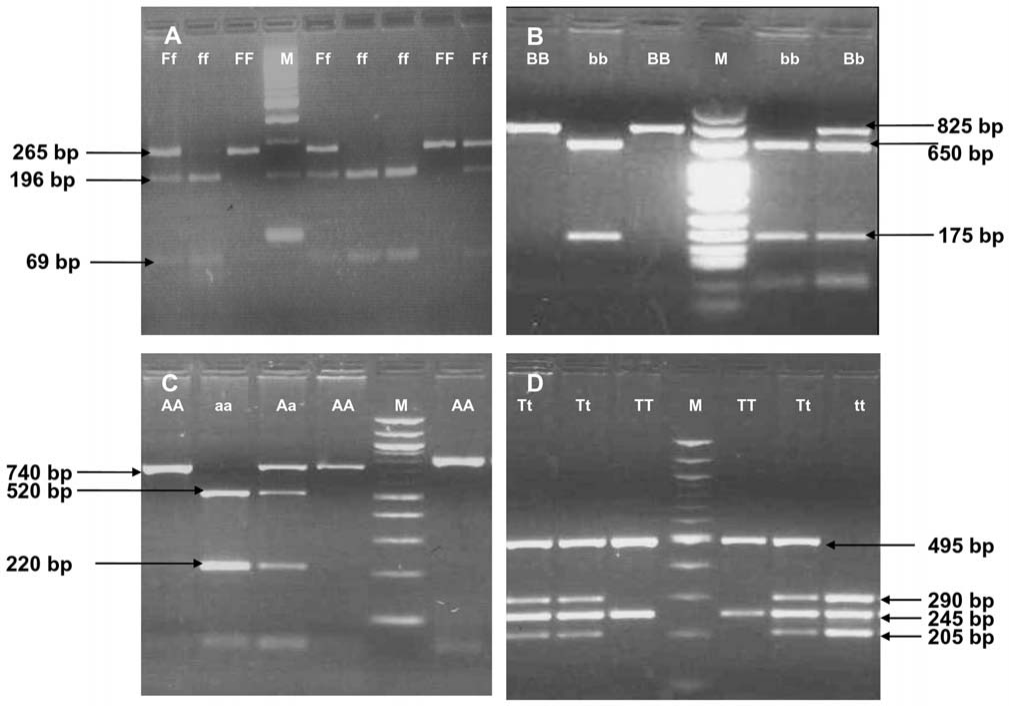
The genotypes for the four SNPs were determined by PCR amplification and restriction digestion of the PCR products with enzymes *FokI, BsmI, ApaI*, and *TaqI*. A: Fok1 digestion: SNP C/T in exon 2 was studied by amplifying a 265 bp fragment using primers 5′AGCTGGCCCTGGCACTGACTCTTGCTCT 3′ and 5′ATGGAAACACCTTGCTTCTTCTCCCTC 3′ with 68°C as annealing temperature and digestion by fok1 at 37°C for 3 hours. Presence of restriction site is denoted by ‘f’ while absence of restriction is denoted by ‘F’. Results show FF (CC) i.e., a 265 bp band or Ff (CT) i.e., 265 bp, 196 bp and 69 bp, bands, ff (TT) i.e., 196 bp and 69 bp bands. M is the100 bp ladder. B: Bsm 1 digestion: SNP A/G in Intron 8 was studied by amplifying an 825 bp fragment using primers 5′CAACCAAGACTACAAGTACCGCGTCAGTGA 3′ and 5′AACCAGCGGGAAGAGGTCAAGGG 3′ with 65°C as annealing temperature and digestion by Bsm1 at 65°C for one hour. Presence of restriction site is denoted as ‘b’ and absence of restriction is denoted by ‘B’. The results show BB (AA) i.e., 825 bp band, Bb (AG) i.e., 825 bp, 650 bp and 175 bp and bands and bb (GG) 650 bp and 175 bp bands. M is 25 bp ladder. C: Apa 1 digestion: SNP T/G in Intron 8 was studied by amplifying an 740 bp fragment using primers 5′ CAGAGCATGGACAGGGAGCAAG 3′ and 5′ GCAACTCCTCATGGCTGAGGTCTCA 3′ with 65°C as annealing temperature and digestion by Apa1 at 37°C for one hour. Presence of restriction site is denoted as ‘a; and absence of restriction is denoted by ‘A’. The results show AA (TT) i.e., 740 bp band, Aa (TG) i.e., 740 bp, 520 bp and 220 bp bands and aa (GG) 520 bp and 220 bp bands. M is 100 bp ladder. D: Taq 1 digestion: SNP T/C in exon 9 was studied by amplifying an 740 bp fragment using primers 5′CAGAGCATGGACAGGGAGCAAG 3′ and 5′GCAACTCCTCATGGCTGAGGTCTCA 3′ with 65°C as annealing temperature and digestion by Taq1 at 65°C for one hour. Presence of restriction site is denoted as ‘t’ and absence of restriction is denoted by ‘T’. The results show TT (TT) i.e., 495 bp and 245 bp bands Tt (TC) i.e., 495 bp, 290 bp, 245 bp and 205 bp bands and tt (CC) 290 bp, 245 bp and 205 bp bands. M is 100 bp ladder.

Table 1 shows the frequencies of *FokI, BsmI, ApaI* and *TaqI* genotypes (figure 1A–D) in the T1D patient group as compared to the control group. *FokI*, *BsmI* and *TaqI* sites were found to be in Hardy Weinberg equilibrium both in patients as well as controls and *ApaI* site was in Hardy Weinberg disequilibrium in both patients and controls. While there were no significant differences in the genotypes of *ApaI* and *TaqI* in patients and controls. *FokI* ‘ff’ was significantly increased in the patient group as compared to controls (p<0.047) and *BsmI* ‘bb’ was significantly decreased in the patient group (p<0.04). However, these differences did not remain significant after Boneferroni's correction.

**Table 1:** Comparison of genotype frequencies of *VDR* SNPs at *Fok1, Bsm1, Apa* and *Taq1* sites in T1D patients with controls.

SNP	T1D		Controls		T1D vs Controls				
	Number N = 236	%	Number N = 197	%	Comparison	p value	Odds Ratio	95% CI	Global p value
FF	142	60.2	116	58.9	FF vs Ff	0.483	1.17	0.774–1.79	0.122
Ff	79	33.5	76	38.6	Ff vs. ff	0.03* ^#^	0.346	0.109–1.086	
ff	15	6.4	5	2.5	FF vs ff	0.06^#^	0.41	0.126–1.246	
	HWE p = 0.38@		HWE p = 0.07		FF vs. Ff+ff	0.862	1.05	0.704–1.58	
					ff vs. FF+Ff	0.047* ^#^	2.6	0.87–8.36	
	N = 236		N = 197						
BB	79	33.5	56	28.4	BB vs Bb	0.735	1.105	0.698–1.75	0.134
Bb	120	50.9	94	47.7	Bb vs bb	0.059	0.638	0.4–1.017	
bb	37	15.7	47	23.9	BB vs bb	0.05*	1.792	0.996–3.23	
	HWE p = 0.59		HWE p = 0.95		BB vs. Bb+bb	0.305	1.3	0.82–1.95	
					bb vs. BB+Bb	0.04*	0.59	0.36–0.99	
	N = 236		N = 197						
AA	85	36.01	60	30.5	AA vs.Aa	0.52	1.17	0.757–1.82	0.09
Aa	133	56.4	110	55.8	Aa vs. aa	0.09	1.81	0.907–3.64	
aa	18	7.6	27	13.7	AA vs. aa	0.04*	2.125	1.02–4.45	
	HWE p = 0.0005		HWE p = 0.04		AA vs Aa+aa	0.263	1.285	0.84–1.96	
					aa vs AA+Aa.	0.056	0.52	0.264–1.02	
	N = 236		N = 197						
TT	91	38.6	80	40.6	TT vs Tt	0.93	0.995	0.651–1.523	0.38
Tt	112	47.5	98	49.75	Tt vs. tt	0.246	0.658	0.335–1.285	
tt	33	14.0	19	9.6	TT vs. tt	0.253	0.655	0.329–1.299	
	HWE p = 0.9		HWE p = 0.16		TT vs. Tt+tt	0.737	0.918	0.612–1.38	
					tt vs. TT+Tt	0.217	1.523	0.805–2.895	

* Corrected p (pc) value is not significant.

#Calculated using Fisher's exact test.

@HWE p value calculated using SHEsis program.

Haplotype analysis was carried out for the four restriction sites studied in the VDR gene in patients and controls using SHEsis program (http://202.120.7.14/analysis/myAnalysis.php) [27]. Additionally, Famhap (http://famhap.meb.uni-bonn.de) was used to confirm the frequencies of the haplotypes. Since both Famhap and SHEsis were giving similar results, we carried out the rest of the analysis using SHEsis only. Table 2 shows the haplotype frequencies of VDR in T1D patients and controls. The software was instructed to drop frequencies less than 0.03 in the analysis. The analysis showed that *FokI* site was in a very weak linkage disequilibium (LD) with *BsmI, ApaI* and *TaqI* with D' values of 0.004, 0.01 and 0.04 respectively. *BsmI*, however, was in strong LD with *ApaI* and *TaqI* with D' values of 0.91 and 0.93 and *ApaI* was also in strong LD with *TaqI* with a D' value of 0.97. Significant differences in terms of haplotypes was observed between patients and controls with a global chi-square of 20.9 for 6 degrees of freedom with a p value of 0.002. Individually, haplotype *FBAt* and *fBAT* were significantly increased in T1D patients (p<0.02 and <0.001 respectively) and *fBAt* was significantly reduced in them as compared to controls (p<0.036).

**Table 2:** Haplotype Analysis for *Fok1, Apa1, Bsm1* and *Taq1* loci using SHEsis software[27]. (http://202.120.7.14/analysis/myAnalysis.php).

	T1D 2N = 472	Controls 2N = 394	T1D Vs Controls			
Haplotype (Nucleotides)	Haplotype Frequency	Haplotype Frequency	χ2	Pearson's p value	Odds Ratio	95% C.I.
*FBAt (CATC)*	0.303	0.233	5.340	0.0208	1.436	1.056–1.953
*FBAT (CATT)*	0.142	0.146	0.032	0.8582	0.966	0.659–1.416
*FbaT (CGGT)*	0.262	0.317	3.311	0.0688	0.759	0.563–1.022
*FbAT (CGTT)*	0.045	0.065	1.611	0.2042	0.683	0.378–1.234
*fBAt (TATC)*	0.063	0.099	4.402	0.0359	0.586	0.354–0.970
*fBAT (TATT)*	0.065	0.021	9.564	0.0019	3.227	1.478–7.049
*fbaT(TGGT)*	0.079	0.074	0.066	0.7976	1.068	0.645–1.768

* Haplotype Frequencies less than 0.03 have not been shown in the analysis.

95% C.I. = 95% confidence interval. Global Chi^2^ = 20.9 with Pearson's p value = 0.002.

Linkage disequilibrium test using SHEsis (D').

D': *Fok1-Bsm1 = 0.004.* *Fok1-Apa1 = 0.010,* *Fok1-Taq1 = 0.04,* *Bsm1-Apa1 = 0.91,* *Bsm1-Taq1 = 0.93,* *Apa1-Taq1 = 0.97.*

### Association of VDR *FokI-BsmI-ApaI-TaqI* Haplotypes with Age at Onset

To study if the VDR haplotypes are associated with age at onset, we divided the patients into two groups adult onset i.e., above 18 and young onset as below or equal to 18 years (Table 3 shows data on 223 samples in whom the exact age at onset was known). The analysis showed that haplotypes *fBAT* anf *FBAt* to be significantly increased in patients who developed diabetes before 18 years of age (p<0.0002 and 0.0077 respectively) and haplotype *FbaT* seemed to be protective from younger onset of T1D (p<0.037). However, adult onset was not significantly associated with any VDR haplotypes in T1D patients.

**Table 3:** Association of VDR haplotypes with age at onset in T1D patients.

VDR Haplotype	*FBAt*	*FBAT*	*fBAT*	*FbaT*	*fBAt*
Nucleotides	*CATC*	*CATT*	*TATT*	*CGGT*	*TATC*
Age at Onset					
Patients≤18 years					
HF* 2N = 268	0.33	0.11	0.08	0.25	0.06
Patients>18 years					
HF 2N = 178	0.27	0.19	0.04	0.27	0.06
Controls					
HF 2N = 394	0.23	0.15	0.02	0.32	0.1
Patients age at onset≤18 years. Vs. Controls					
p value	0.0077	0.17	0.0002	0.0367	0.082
Odds Ratio	1.6	0.718	4.16	0.69	0.59
95% CI	1.13–2.27	0.45–1.15	1.84–9.4	0.483–0.99	0.33–1.08
Patients age at onset >18 years. Vs. Controls					
p value	0.274	0.21	0.112	0.273	0.169
Odds Ratio	1.26	1.35	2.2	0.8	0.62
95% CI	0.84–1.89	0.84–2.17	0.81–5.93	0.54–1.19	0.31–1.23

Adult onset was considered above 18 years of age and child onset as 18 years or below 18 years. The two groups were compared with controls using SHEsis software.

Linkage Disequilibrium tests for patients below 18 years of age (SHEsis).

D': *Fok1-Bsm1* = 0.048, *Fok1-Apa1* = 0.005, *Fok1-Taq1* = 0.054, *Bsm1-Apa1* = 0.914, *Bsm1-Taq 1* = 0.949, *Apa1-Taq1* = 0.955.

Linkage Disequilibrium tests for patients above 18 years of age (SHEsis).

D': *Fok1-Bsm1* = 0.004, *Fok1-Apa1* = 0.009, *Fok1-Taq1* = 0.104, *Bsm1-Apa1* = 0.886, *Bsm1-Taq 1* = 0.93, *Apa1-Taq1* = 0.946.

### Association of VDR *FokI-BsmI-ApaI-TaqI* Haplotypes with Gender and Age at Onset of T1D Patients

To study if this association of VDR haplotypes had any gender bias, comparisons were done between female/male patients and female/male controls and also with all controls (female + males). Table 4 shows the gender-wise analysis of VDR haplotype using SHEsis software. While *fBAT* was significantly increased in female patients as compared to controls (both female controls and all controls), *FBAt* was significantly increased in males as compared to controls (both male controls and all controls). *FbaT* was significantly reduced in female patients when compared to female controls and *fBAt* was significantly reduced in male patients as compared to controls. A comparison of female patients with male patients did not reveal any significant differences. Since the patients with younger age at onset were showing significant differences, we analysed the data with respect to age at onset in the two genders (data not shown). The haplotypes *FBAt* and *fBAT* were significantly increased in male patients below the age at onset of 18 years as compared to controls (p<0.0024 and p<0.0017 respectively) and *FBAT* was significantly reduced (p<0.0038) in the male patients below the age at onset of 18 years as compared to controls. No significant differences were observed in the *VDR* haplotypes of male patients with age at onset above 18 years when compared to controls. However, in the females, the haplotype *fBAT* was significantly increased in patients irrespective of their age at onset whether early or adult onset and haplotype *FbaT* was significantly reduced in female patients with less than 18years age at onset.

**Table 4:** Comparisons of frequencies of VDR haplotypes in male and female patients with male and female controls, all controls and between male and female patients using SHEsis software.

VDR Haplotype	*FBAt*	*FBAT*	*fBAT*	*FbaT*	*fBAt*
Nucleotides	*CATC*	*CATT*	*TATT*	*CGGT*	*TATC*
Female (HF)*Patients 2N = 210	0.267	0.173	0.084	0.252	0.067
Male (HF)Patients 2N = 262	0.333	0.119	0.048	0.268	0.056
Female (HF)Controls 2N = 162	0.225	0.166	0.012	0.348	0.114
Male (HF)Controls 2N = 232	0.231	0.128	0.030	0.314	0.096
All (HF)Controls 2N = 394	0.233	0.146	0.021	0.317	0.099
Female Patients vs. Controls					
p value	0.41	0.42	0.0003	0.074	0.17
Odds Ratio	1.18	1.21	4.21	0.71	0.64
95% C.I.	0.8–1.74	0.76–1.9	1.81–9.8	0.48–1.03	0.34–1.21
Female Patients vs. Female Controls					
p value	0.42	0.95	0.0025	0.0298	0.095
Odds Ratio	1.22	1.02	7.25	0.61	0.54
95% C.I	0.75 –1.98	0.59–1.77	1.64–32.1	0.39–0.95	0.26–1.12
Male Patients vs. Controls					
p value	0.004	0.33	0.051	0.185	0.052
Odds Ratio	1.67	0.33	0.051	0.185	0.052
95% C.I	1.18–2.37	0.49–1.27	0.97–5.8	0.56–1.12	0.29–1.01
Male Patients vs. Male Controls					
p value	0.01	0.75	0.29	0.27	0.09
Odds Ratio	1.69	0.75	0.29	0.27	0.09
95% C.I.	1.13–2.54	0.54–1.57	0.64–4.27	0.54 –1.19	0.28–1.11
Female Patients vs. Male Patients					
p value	0.089	0.113	0.13	0.61	0.67
Odds Ratio	0.71	1.5	1.77	0.61	0.67
95% C.I.	0.47–1.06	0.9–2.56	0.84 –3.75	0.59–1.36	0.55– 2.52

HF = Haplotype frequency.

Linkage Disequilibrium tests for Female patients using SHEsis software.

D': *Fok1-Bsm1* = 0.066, *Fok1-Apa1* = 0.042, *Fok1-Taq1* = 0.048, *Bsm1-Apa1* = 0.9, *Bsm1-Taq 1* = 0.966, *Apa1-Taq1* = 0.946.

Linkage Disequilibrium tests for Male patients using SHEsis software.

D': *Fok1-Bsm1* = 0.015, *Fok1-Apa1* = 0.03, *Fok1-Taq1* = 0.014, *Bsm1-Apa1* = 0.898, *Bsm1-Taq 1* = 0.923, *Apa1-Taq1* = 0.957.

### Gene to Gene Interaction of *VDR* Haplotypes with Predisposing *HLA* Alleles

Based on the SHEsis and Famhap analysis, haplotypes were constructed manually for each sample. The haplotype with the highest frequency as calculated by SHEsis was constructed from the genotype of each sample and the second haplotype was constructed by subtraction of the first haplotype. Simultaneous presence of different haplotypes with the predisposing *HLA* alleles DRB1*0301, DRB1*0401, DRB1*0402 and DRB1*0405 (listed as *DR3* for the sake of simplicity in Table 5 which includes all these alleles) in patients was studied. Interestingly, haplotypes *FBAT, FbaT*, whose frequencies were not significantly different in patients and controls, showed a significant increase in patients when present along with the predisposing *DRB1* alleles while the same haplotypes were protective when associated with non-predisposing alleles of *DRB1*. Similar results were obtained with other haplotypes like *FBAt, fBAT and fbaT* in association with the predisposing *HLA-DRB1* alleles, as shown in table 5.

**Table 5:** Simultaneous presence of different VDR haplotypes along with predisposing HLA-DRB1*0301, *0401, *0402 and *0405 alleles.

*VDR* Haplotype	T1D (N = 233)		Controls (N = 191)		T1D vs CONTROLs		
-*HLA-DR3* +/−	No.	%	No.	%	p Value	OR	95% CL
*FBat-DR3-ve#*	0	0.00	1	0.52	0.45	0.27	0.007–3.0
*FBaT-DR3+ve*	3	1.29	0	0.00	0.16	5.9	0.7–58.8
*FBaT-DR3-ve*	1	0.43	3	1.57	0.241	0.34	0.07–1.5
*FBAt-DR3+ve*	107	45.92	20	10.47	<10^−6^ *	7.2	4.15–12.8
*FBAt-DR3-ve*	27	11.58	60	31.41	<10^−6^ *	0.29	0.16–0.49
*FBAT-DR3+ve*	48	20.60	7	3.66	<10^−6^	6.8	2.9–16.9
*FBAT-DR3-ve*	4	1.72	34	17.80	<10^−8^ *	0.07	0.03–0.2
*FbaT-DR3+ve*	103	44.21	24	12.57	<10^−6^ *	5.5	3.25–9.39
*FbaT-DR3-ve*	16	6.87	105	54.97	<10^−6^ *	0.05	0.03–0.09
*FbAt-DR3+ve*	1	0.43	0	0.00	0.5	2.5	0.23–848.2
*FbAt-DR3-ve*	0	0.00	1	0.52	0.45	0.27	0.007–3.0
*FbAT-DR3+ve*	14	6.01	3	1.57	0.02**	3.7	1.5–9.6
*FbAT-DR3-ve*	3	1.29	18	9.42	0.0003*	0.12	0.04–0.46
*fBaT-DR3+ve*	3	1.29	1	0.52	0.4	1.9	0.45–14.5
*fBaT-DR3-ve*	0	0.00	1	0.52	0.45	0.27	0.007–3.0
*fBAt-DR3+ve*	14	6.01	9	4.71	0.7	1.3	0.5–3.3
*fBAt-DR3-ve*	1	0.43	29	15.18	<10^−8^ *	0.03	0.01–0.1
*fBAT-DR3+ve*	34	14.59	7	3.66	0.0003*	4.5	1.8–11.4
*fBAT-DR3-ve*	7	3.00	13	6.81	0.11	0.42	0.15–1.2
*fbaT-DR3+ve*	29	12.45	0	0	<10^−8^ *	55.2	8.2–173.4
*fbaT-DR3-ve*	8	3.43	9	4.71	0.67	0.72	0.25–2.1
*fbAt-DR3+ve*	3	1.29	0	0.00	0.16	6	0.7–58.8
*fbAt-DR3-ve*	1	0.43	1	0.52	0.7	0.81	0.13–5.1
*fbAT-DR3+ve*	7	3.00	2	1.05	0.15	2.6	0.9–8.2
*fbAT-DR3-ve*	1	0.43	8	4.19	0.009*	0.13	0.04–0.5

No. shows the number of individual positive for the indicated VDR haplotype and *DR3* allele.

#DR3+ve includes all the Predisposing Alleles i.e. DRB1*0301,*0401,*0402 and *0405.

* Corrected P(pc) value is significant.

** Corrected P(pc) value is not significant.

These results prompted us to study the interaction between two unlinked loci i.e., *VDR* and the predisposing *HLA-DRB1* alleles since it was crucial to study the difference in LD patterns of these two unlinked loci between patients and controls. For this kind of analysis we used LD based statistics as described by Zhao et al (22). The analysis has been shown in Table 6. Two most frequent haplotypes of *VDR, FbaT* and *FBAT* and a relatively less frequent haplotype in patients *fbaT* do not show any significant difference between patients and controls when studied by themselves. However, when studied in association with the predisposing HLA-DRB1*0301, *0401, *0402 and *0405 collectively (listed as DR3 in tables 5–7) in T1D patients, these VDR haplotypes show significant linkage disequilibrium. Since all these haplotypes had consistently *T* allele at *TaqI* site and either of the two alleles at *FokI, BsmI* and *ApaI* sites, in order to decipher which alleles were actually involved, we studied the LD pattern between the predisposing *HLA-DR3* with each of these alleles at the four SNP sites separately.

**Table 6 pone-0008023-t006:** LD based statistics to study the linkage disequilibrium between two unlinked loci (VDR haplotypes and predisposing HLA-DRB1*0301, *0401, *0402 and *0405 alleles shown collectively as DR3 in the table 6).

*VDR haplotype-DR3* #	T1D (N = 233) δ_A_	Controls (N = 191) δ_N_	V_A_	V_N_	T1	p value
*FBAT-DR3*	0.035	0.0056	0.00004	0.000035	11.52	0.0006
*FBAt-DR3*	0.042	0.03	0.0001	0.000075	0.82	0.365
*FbaT-DR3*	0.0576	0.0233	0.000081	0.000069	7.84	0.005
*fBAT-DR3*	0.0222	0.01	0.0000323	0.00003	2.39	0.122
*fBAt-DR3*	0.01	0.013	0.000016	0.000043	0.15	0.698
*fbaT-DR3*	0.0146	−0.0028	0.000032	0.00000085	9.195	0.002

# *DR3+ve* includes all the Predisposing Alleles i.e. DRB1*0301,*0401,*0402 and *0405.

δ_A_, δ_N_, V_A_, V_N_ and T1 calculated as shown in statistical methods.

Frequencies of *DR3* = 0.6, *FBAT* = 0.12, *FBAT-DR3* = 0.107, *FBAt* = 0.33, *FBAt-DR3* = 0.24, *FbaT* = 0.264, *FbaT-DR3* = 0.216, *fBAT* = 0.088, *fBAT-DR3* = 0.075, *fBAt* = 0.034, *fBAt-DR3* = 0.03, *fbaT* = 0.079, *fbaT-DR3* = 0.062 in patients and *DR3* = 0.107, *FBAT* = 0.118, *FBAT-DR3* = 0.0183, *FBAt* = 0.233, *FBAt-DR3* = 0.055, *FbaT* = 0.369, *FbaT-DR3* = 0.063, *fBAT* = 0.052, *fBAT-DR3* = 0.0157, *fBAt* = 0.099, *fBAt-DR3* = 0.0236, *fbaT* = 0.026 and *fbaT-DR3* = 0 in controls. Frequencies of the *VDR* haplotypes are marginally different from Table 2 as these have been calculated from the reconstructed haplotypes based on SHEsis program to study interaction with predisposing *HLA* alleles, whereas Table 2 shows the frequencies as calculated by SHEsis analysis.

**Table 7:** LD based statistics to study the linkage disequilibrium between two unlinked loci (VDR alleles F/f, B/b, A/a and T/t and predisposing HLA-DRB1*0301, *0401, *0402 and *0405 alleles shown collectively as DR3 in the table).

*VDR allele-DR3* #	T1D (N = 233) δ_A_	Controls (N = 191) δ_N_	V_A_	V_N_	T1	p value
*F-DR3*	0.063	0.022	0.00011	0.000016	13.3	0.0002
*f-DR3*	0.042	0.0245	0.000078	0.000069	2.08	0.149
*B-DR3*	0.071	0.03	0.00012	0.000058	9.497	0.002
*b-DR3*	0.07	0.0136	0.00011	0.000062	17.84	0.00002
*A-DR3*	0.075	0.031	0.00012	0.000046	11.66	0.0006
*a-DR3*	0.058	0.021	0.0001	0.00006	8.65	0.003
*T-DR3*	0.0696	0.024	0.00012	0.000039	13.07	0.0003
*t-DR3*	0.049	0.029	0.00011	0.000073	2.29	0.13

# *DR3+ve* includes all the Predisposing Alleles i.e. DRB1*0301,*0401,*0402 and *0405.

δ_A_, δ_N_, V_A_, V_N_ and T1 calculated as shown in statistical methods.

Frequencies of *DR3* = 0.6, *F* = 0.76, *F-DR3* = 0.519 *f* = 0.23, *f-DR3* = 0.18, *B* = 0.59, *B-DR3* = 0.425, *b* = 0.41, *b-DR3* = 0.315, *A* = 0.642, *A-DR3* = 0.46, *a* = 0.358, *a-DR3* = 0.273, *T* = 0.624, *T-DR3* = 0.444, *t* = 0.376, *t-DR3* = 0.275 in patients and *DR3* = 0.107, *F* = 0.78, *F-DR3* = 0.105, *f* = 0.21, *f-DR3* = 0.047, *B* = 0.52, *B-DR3* = 0.086, *b* = 0.48, *b-DR3* = 0.065, *A* = 0.59, *A-DR3* = 0.094, *a* = 0.411, *a-DR3* = 0.065, *T* = 0.66 *T-DR3* = 0.0942, *t* = 0.34, *t-DR3* = 0.065 in controls. Frequencies of the *VDR* alleles are marginally different from Table 1 depending on which samples were typed for all the four loci and *HLA-DRB1* in both patients and controls.

Table 7 shows the LD pattern of the *FokI, BsmI, ApaI* and *TaqI* alleles with predisposing HLA-DRB1*0301, *0401, *0402 and *0405 (listed as DR3 in tables 5–7) in T1D patients. *F* and *T* alleles in the exons 2 and 9 for *FokI* and *TaqI* restriction sites respectively show significant interactions (p<0.0002 and 0.0003 respectively). However, for *BsmI* and *ApaI* sites both the alleles were significantly associated with predisposing HLA alleles suggesting their null effect.

### Sequence Analysis of HLA DRB1*0301 Promoter Region

**Figure 2:**
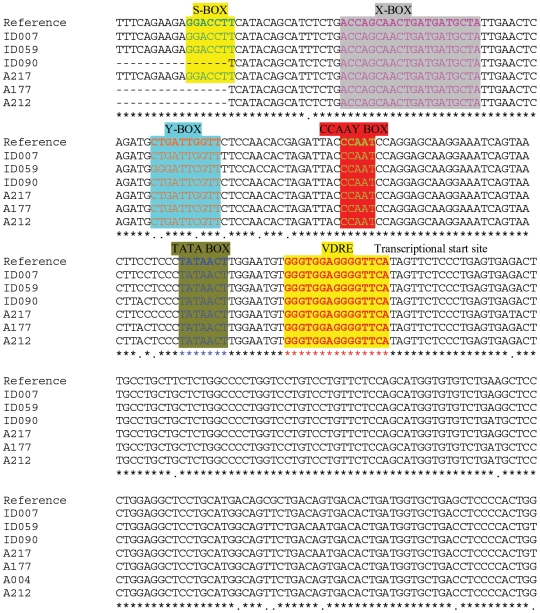
Promoter region of HLA- DRB1 was sequenced from 3 subjects suffering from type 1 diabetes and 3 normal healthy individuals homozygous for DRB1*0301. The sequence showing important regulatory elements like S-box, X-box, Y-box, CCAAY-box, TATA-box and VDRE are highlighted. Stars (*) in the last row show homology and dots (.) show nucleotide substitution in one or more samples at that particular site and dashes(-) represent gaps inserted to maximize the homology. The ID and A numbers represent the T1D and control samples sequenced respectively. The sequences have been aligned with reference sequence gi|545423|gb|S69987.1| HLA-DRB1 (DRB1*0301) {promoter} [human, lymphoblastoid cell line AVL, Genomic, 416 nt] [64].

Since 85.9% of the patients had DRB1*0301, to study if the interaction of *VDR* with predisposing *HLA* alleles was mediated by VDRE present in the promoter region of the allele, we amplified the promoter region of 3 T1D subjects and 3 healthy controls homozygous for HLA-DRB1*0301. Sequencing of the amplified promoter region was done to determine the VDRE variants in the North Indian population. Sequences were aligned using ClustalW2, and the presence of a VDRE was confirmed in-silico using JASPAR_CORE version 3.0 database using default conditions [28]. Figure 2 shows the HLA- DRB1*0301 promoter sequences showing the localization of vitamin D response element (VDRE) in the promoter region of HLA-DRB1*0301 from the 6 subjects. Important regulatory elements like S-box, X-box, Y-box, CCAAY-box, TATA-box and VDRE are highlighted in the figure. Interestingly, the alignment showed exactly the same sequence of VDRE in the promoter region of HLA-DRB1*0301 which has been shown to influence the expression of *HLA* allele DRB1*1501 recently by Ramagopalan et al [26] suggesting the bases for interaction of VDR with HLA-DRB1*0301.

### Altered Expression of HLA-DRB1*0301 by 1,25-(OH)_2_D_3_ (Calcitriol)

#### Flow cytometry

**Figure 3:**
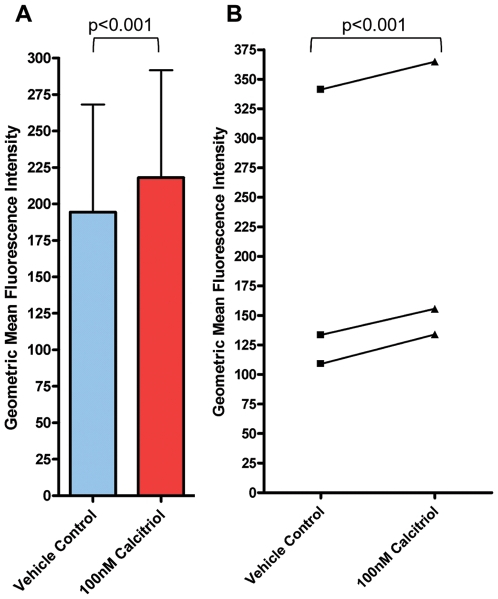
Flow cytometric analysis of HLA-DR expression. B-LCLs VAVY and DUCAF cells were treated with 100 nM calcitriol or equal volume of alcohol as vehicle control. Both VAVY and DUCAF cells show a significant increase in surface HLA-DR expression as determined by the geometric mean flurescence intensity of staining with HLA-DR-PE antibody. A. The figure shows mean±S.E.M. of three independent experiments. Two tailed Paired T test shows the difference to be significant with p<0.001 i.e., enhanced expression of HLA-DR in B-LCLs treated with calcitriol as compared to untreated ones. B: Line graph showing the extent of enhanced HLA-DR expression in three independent expeiments.

HLA-DRB1*0301 homozygous B-lymphoblastoid cell lines (B-LCL) VAVY (International Histocompatibility Workshop cell line Number IHW09023) and DUCAF (International Histocompatibility Workshop cell line Number IHW09019) were treated with 100 nM of calcitriol (Sigma) for 24 hours and stained with anti-HLA DR-PE antibody (BD Biosciences) to study the expression of HLA-DR on B-LCL cells treated with or without calcitriol and acquired on BD-LSR flowcytometer. The data was analysed using WinMDI 2.9 software. The results showed 1.2-1.3 fold higher expression of HLA-DR in the B-LCLs treated with calcitriol as compared to the vehicle controls in three independent experiments (Figure 3). Two tailed Paired T test shows the difference to be significant with p<0.001 suggesting enhanced expression of HLA-DR in B-LCLs treated with calcitriol as compared to untreated ones.

### Real Time PCR

RNA was extracted from the B-LCLs VAVY and DUCAF after 24 hour treatment with calcitriol and vehicle control, reverse transcribed and real time PCR was performed for HLA-DR to study its expression. The data shows an average of 1.79±0.28 (mean±S.D. of three independent experiments) fold increase in the *HLA-DRB1* transcripts from B-LCL treated with calcitriol as compared to the vehicle control. We also confirmed these results using peripheral blood mononuclear cells (PBMCs) derived from a normal healthy control homozygous for HLA-DRB1*0301 and observed a 1.77 fold increase in expression of HLA-DR when treated with calcitriol as compared to untreated PBMCs.

## Discussion

In this study we show that the *F* and the *T* alleles of *FokI* and *TaqI* site of *VDR* interact with predisposing HLA DRB1*0301 through VDRE present in the promoter region of the DRB1*0301 allele. VDR-1,25-(OH)_2_D_3_ complex has been shown to play a significant role in interfering with the signaling of transcription factors (like NFAT, NF-κB and AP-1) involved in the regulation of immunomodulatory genes [6], [29]–[33] as well as expression of HLA class-II alleles on monocytes and human bone cells [13], [14]. Polymorphisms in the *VDR* gene have been shown to influence *VDR* mRNA and protein levels [34], which in turn may influence the immunomodulatory function of VDR. We have studied four single nucleotide polymorphisms (*FokI, BsmI, ApaI* and *TaqI* sites) in the *VDR* region in T1D and compared them to normal healthy controls. The SNP detected by *FokI* digestion is the only polymorphisms at the translation start site where a ‘*T*’ to ‘*C*’ substitution alters the VDR protein in such a way that there are either two *ATG* start sites separated by six nucleotides or only one start codon if the ‘*T*’ is substituted by ‘*C*’. The former gives rise to a restriction site for *FokI* and is designated as ‘*f*’ allele which encodes a 427 amino acid protein, while the latter is designated as ‘*F*’ allele encoding a 424 amino acid protein. [35]–[37]. The shorter ‘*F*’ allele has been reported to have emerged later after the divergence of hominids from Apes [38] and a higher percentage of the allele suggests an evolutionary advantage [39]. Recently, ‘*F*’ allele has also been shown to affect the immune system with a more active immune system for short ‘F’-VDR suggesting its role in the immune-mediated diseases [40]. The gene to gene interaction analysis shows allele *F* and predisposing *MHC* alleles to have significant interaction suggesting the integrated roles of the MHC alleles in antigen presentation and *VDR-F* allele in enhanced autoimmune responses since shorter *F-VDR* has been shown to result in higher NF-kappaB- and NFAT-driven transcription as well as higher IL-12p40 promoter-driven transcription [40].

In the present study, we observed homozygous ‘*bb*’ to be significantly reduced in the patients in contrast to a report by McDermott et al [41] in Southern Indian families where ‘*b*’ allele was shown to be excessively transmitted to the affected offsprings. This difference could be due to different ethnicity of south and north Indian populations since the *B/b* polymorphism does not have any functional significance. Individual *VDR* polymorphisms have been studied in different populations with conflicting reports. Some reports find a positive association of *VDR* polymorphism with T1D [41]–[46], while there are a considerable number of reports where no association of *VDR* polymorphism with T1D was observed [47]–[49]. In a meta-analysis Guo et al [50] did not see any evidence of significant association between *VDR* polymorphism and T1D in either case-control or family transmission. Yet another meta-analysis [51] concluded that *F* and the *B* alleles at *FokI* and *BsmI* site showed an increased relative risk for T1D as regional winter UVR levels increased, however, the association of *TaqI T* allele with T1D decreased with winter suggesting that the environmental UVR conditions may influence the association between *VDR* genotype and T1D risk. Hence, the contradictory reports in the literature with respect to *VDR* associations in T1D could be due to ethnic differences in the frequencies of different SNPs in different populations, the environmental UVR and the pleiotropic behaviour of *VDR*.

It is important that all the SNPs of the gene constituting a haplotype be studied as representative of the allele or the so-called “pseudo-allele” as such rather than individual SNPs since it is possible that the 5′ and 3′ polymorphisms may be functionally linked to each other either through their independent influences on VDR activity or by physical interaction [52]. SHEsis and Famhap analysis showed *BsmI* site was in strong LD with *ApaI* site and *TaqI* site with D' values of 0.91 and 0.93 and *ApaI* site was also in strong LD with *TaqI* site with a D' value of 0.97, however, the *FokI* site was in a very weak linkage disequilibium (LD) with *BsmI, ApaI* and *TaqI* sites with D' values of 0.004, 0.01 and 0.04 respectively. The haplotypes, with *FokI* alleles, however, could still be constructed because 60.2% of the patients had *FF* genotype and 6.4% had *ff* genotype. SHEsis analysis in the present study showed haplotype ‘*FBAt*’ and ‘*fBAT*’ to be significantly increased in patients as compared to controls. Both these haplotypes were increased in patients with less than 18 years as age at onset. However, there was no significant difference in patients with adult age at onset as compared to controls. We also observed a gender-wise distribution of these haplotypes. While *‘fBAT*’ was significantly increased in female patients as compared to all controls and female controls, ‘*FBAt*’ was significantly increased in male patients as compared to all controls and male controls. To study the distribution of these haplotypes in the two genders with the age at onset, we divided the males and females into two groups each i.e., with adult onset (above 18 years) and younger onset (≤18 years). Interestingly, both ‘*FBAt*’ and ‘*fBAT*’ were significantly increased in male patients with younger age at onset and not in the male patients with adult onset of T1D. However, ‘*fBAT*’ was significantly increased in female patients in both adult onset as well as younger onset of the disease. These data suggest that different haplotypes with ‘*F*’ or ‘*T*’ allele may be involved in differential effects on the immune systems in males and females patients since both *FokI* and *TaqI* sites have been implicated in transcription of *VDR* [34].

We have shown earlier that HLA-DRB1*0301, *0401 and *0405 are predisposing for T1D [2]. Association of *MHC* class-II alleles with an autoimmune disease could be due to its antigen presenting function and *VDR* may have a role in regulation of autoimmune responses through VDR −1,25 (OH)_2_D3 complex. Hence, we sought to study whether interaction of two independently assorting genes i.e. predisposing *HLA* alleles and *VDR* alleles have a role to play in the precipitation of the disease. T1D is a complex, multi-factorial disease where individual factors may not show any significant difference, however, when studied in association with other predisposing factors, integration of different factors may be implicated. To study the interaction of two genes which are not linked i.e., *VDR* (on chromosome 12q 13–14) and predisposing *HLA* alleles (on chromosome 6p21.3), we used “LD based statistics” as described by Zhao et al [25]. We tested the interaction by comparing the difference in the LD levels between *VDR* haplotypes and HLA-DR3 (HLA-DRB1*0301, *0401, *0402 and *0405 collectively) between cases and controls. Since there is a possibility of background LD between the two unlinked loci in the population, we used more robust case-control formula as Zhao et al [25] suggest that case-only formula may lead to type 1 error. Moreover, Zhao et al [25] argue that LD based statistics has much higher power in detecting interaction than does the logistic regression. The analysis revealed *FBAT-DR3*, *FbaT-DR3* and *fbaT-DR3* to show significant interactions in the patients as compared to controls. It is obvious from these data that ‘*T*’ allele is common in all the haplotypes which showed interaction with pre-disposing *HLA* alleles, while haplotypes with both ‘*F*’ and ‘*f*’ alleles were associated with *HLA-DR3* allele, so to dissect out which of these alleles were actually interacting with *DR-3* allele, we applied LD based statistic to individual *Fok, BsmI, ApaI* and *Taq 1* alleles (Table 7) and the data suggested that ‘*F*’ allele at *FokI* site and ‘T’ allele at the *TaqI* site show significant interaction with the predisposing *HLA-DR3*. However, both ‘*B*’ and ‘*b*’ alleles and ‘*A*’ and ‘*a*’ alleles at *BsmI* and *ApaI* sites respectively showed interaction suggesting their null effect.

An association of *VDR* genotypes with *VDR* mRNA and VDR protein has been demonstrated [21], [34], [53] in peripheral blood mononuclear cells providing functional relevance to the *VDR* polymorphisms. *FokI* and TaqI genotypes were observed to be independent determinants of insulin secretion and *VDR* mRNA and protein levels [34]. Gross et al [53] showed an increased transcription rate of the *VDR* gene in cells with ‘*FF*’ genotype. ‘*F*’ allele which encodes the shorter protein has been shown to be transcriptionally 1.5 to 2.5 fold more active than ‘*f*’ allele and has also been shown to associate more avidly with Transcription factor II B (TF IIB) [39]. Interestingly, Jurutka et al [39] show that vitamin-D3 mediated transcription requires specific physical interaction of VDR with TF IIB which involves both C and N terminal domains in the receptor. Moreover, while *FokI* site is situated in the N-terminal end of the VDR molecule, the ligand binding domain is situated in its C-terminal [41], which explains why *FokI* and *TaqI* sites may be playing detrimental roles in the manifestation of T1D in the presence of predisposing *MHC* alleles. *FokI* and *TaqI* genotypes have been shown to contribute to *VDR* mRNA and protein levels independently[54]. While the promoter has been shown to regulate production of mRNA, the 3′UTR is involved in stability/degradation of mRNA, and their interaction or combined effects may regulate the net availability of the mRNA for translation into the VDR protein [54]. Hence, *F* allele of *FokI* and *T* allele of *TaqI* along with predisposing MHC alleles play an integrated role in the autoimmune responses in T1D.

Functionally, *VDR-FF* individuals have been shown to produce more IL-12p40 and IL-12p35 and even the IL-12 protein is higher in these individuals as compared to those with *ff* genotype [40]. On the other hand, the VDR ligand 1,25 (OH)_2_D_3_ has been shown to inhibit lymphocyte proliferation and production of IFN-gamma, IL-2 and IL-12 [54]. Hence, in the present scenario, while predisposing MHC alleles may be involved in auto-antigen presentation, the VDR haplotypes with ‘*F*’ and ‘*T*’ alleles may have a role in increased amount of VDR protein resulting in increased IL-12 production which in turn may lead to other downstream pro-inflammatory immune responses against auto-antigens in T1D. And administration of 1,25-(OH)_2_D_3_ in type 1 diabetes may be able to inhibit these pro-inflammatory immune responses by binding to VDR which induces conformational changes in VDR, promoting its heterodimerization with Retinoid X Receptor (RXR), followed by translocation of this complex into the nucleus which in turn binds to the vitamin D_3_ responsive elements (VDRE) or the DNA binding domains in promoter regions of 1,25-(OH)_2_D_3_ responsive genes [4], thereby down regulating transcription of the pro-inflammatory cytokines like IL-2, IFN-γ and IL-12 [7]–[9], [11], thus delaying the pathogenesis of type 1 diabetes.

Additionally, presence of VDRE in the promoter region of the predisposing HLA-DRB1*0301 may also have a significant role in manifestation of the disease since a direct interaction between *HLA-DRB1* and vitamin D has been demonstrated recently [26]. VDRE is shown to have subtle sequence differences and different *HLA* alleles may have different VDRE sequences in their promoter regions influencing the expression of HLA alleles [26]. Gene expression of vitamin D regulated genes has been shown to be influenced by these subtle sequence differences within the classical VDRE [6]. Hence, we sequenced the promoter region of normal and affected individuals homozygous for HLA-DRB1*0301 and discovered that VDRE sequence observed in DRB1*0301 promoters from North Indians is homologous to the VDRE sequence which had been shown to up-regulate the expression of HLA DRB1*1501 allele in Multiple Sclerosis [26]. Ramagopalan et al [26] showed that the VDRE corresponding to the Multiple Sclerosis (MS) associated DRB1*1501 haplotype bound to recombinant VDR/RXR with high specificity in vitro in contrast to relatively lower affinity for the VDRE variants observed in the non-MS associated *HLA-DRB1* haplotypes which were not responsive to vitamin D3 [26]. 85.9% of the patients in the present study had HLA-DRB1*0301 with 27.7% being homozygous and the rest heterozygous with DRB1*04 or any other alleles (data not shown). These data suggested that the interaction of *VDR* with DRB1*0301 is through the VDRE present in the promoter region of the HLA allele. Interestingly, both Multiple Sclerosis and T1D have *HLA* as the main susceptibility locus and vitamin D a strong environmental element since supplementation of 1,25-(OH)_2_D_3_ analog in NOD mice before the onset of Insulitis, has been effectively shown to prevent the disease progression. However, this treatment was ineffective when Insulitis had already been established [15]–[18]. Similarly, in humans, vitamin D supplementation in early childhood has been shown to reduce the incidence of T1D [19]. To study whether the interaction of VDR with the HLA DRB1*0301 is through VDRE present in the promoter region of the HLA-DRB1*0301, we stimulated the International Histocomopatibility Workshop's DRB1*0301 homozygous B-lymphoblastoid cell line VAVY (B-LCL IHW09023) and DUCAF (B-LCL IHW09019) with 100 nM of calcitriol and checked for the expression of DR using flow cytometry and real time PCR. The B-LCLs showed 1.2–1.3 fold enhanced expression on flow cytometry, probably because of constitutively high levels of HLA- DR expression on the cell lines tested. However, this enhanced difference was statistically significant on two tailed paired T test (p<0.001). Ramagopalan et al [26] have also shown 1.3 fold increase in expression of HLA-DRB1 in DRB1*15 homozygous cell line PGF on addition of calcitriol and they too found it to be statistically significant. Interestingly, however, real time PCR showed 1.8±0.28 fold increase in the HLA-DR transcripts. Thus, the enhanced expression of HLA-DR on the B-LCLs stimulated with calcitriol as compared to the unstimulated one confirms that indeed the interaction of VDR with HLA-DRB1*0301 is occurring through the VDRE present in the promoter region of the gene. Based on the earlier studies and the present data one can speculate that in the absence of required amount of Vitamin D in early life in the predisposed individuals with HLA-DRB1*0301, the expression of the allele may be impaired in the thymus [26] resulting in escape from thymic deletion of autoreactive T cells leading to T1D manifestations. We have shown earlier that simultaneous presence of DRB1*0301 along with homozygous *INS-VNTR* class-I was significantly increased (p<10^−8^) in T1D patients, giving a relative risk of 70.81 [2]. *INS-VNTR* class-I has also been shown to be associated with lower expression of Insulin in thymii of fetuses as compared to Class-III alleles [55], [56] which may be responsible for poor thymic education for insulin resulting in autoimmunity against pancreatic beta cells. The present study provides additional evidence based on the statistically significant interaction between the predisposing *HLA* allele and high producer alleles of *VDR* which may be detrimental for the manifestation of T1D in the absence of 1,25-(OH)_2_D_3_ in early childhood and/or *in-utero* and this interaction is mediated by VDRE present in the promoter region of DRB1*0301.

## Materials and Methods

Genomic DNA was extracted using a standard protocol from 10 ml of blood from T1D patients and healthy controls from the same ethnic background after obtaining informed written consent and Institutional Human Ethics Committee's approval from both All India Institute of Medical Sciences (AIIMS) and National Institute of Immunology (NII).

236 T1D subjects and 197 normal healthy controls based in Delhi, originally from three states of North India, Uttar Pradesh, Haryana and Punjab, were studied for *VDR* polymorphisms. The patients studied in the current study were recruited from ‘Type 1 Diabetes Clinic’ at All India Institute of Medical Sciences, New Delhi, India, in a consecutive manner from 2004–2008. Most of these patients were part of our earlier studies [3], [57] and were recruited based on their availability in follow up in the clinic and informed consent and were representative of a general population of T1D patients in North India. All the patients were carefully assessed (by RG) and categorized as type 1, type 2 and fibrocalculous pancreatopathy according to the recent classification of the American Diabetes Association expert committee [58]. All of the subjects included in the study required insulin for glycemic control. Insulin requiring patients with fibrocalculous pancreatopathy and subjects with diabetes in whom glycemic control was achieved with diet and oral anti-diabetic drug were excluded from the study as described earlier [3]. The T1D group consisted of 105 females with the mean age at onset 14.74±7.57 and 131 males with a mean age of onset 16.89±7.25. The control group consisted of 81 females and 116 males with a mean age of 30.1±10.2.

Normal healthy controls from the same ethnic background with no history (of self or family) of any autoimmune or infectious diseases were included in the study. They represent the same source population because the controls too, like the patients, belonged to three states of North India, Uttar Pradesh, Haryana and Punjab. The controls were the students, scholars and employees of NII and AIIMS who belonged to these three states. They were not selected in particular but were random individuals who gave informed consent to draw blood and did not have any history of infectious or autoimmune diseases. Controls were not clinically tested; however, they were asked if they had any infectious, autoimmune or any other disease. Only healthy individuals with no disease, symptoms of a disease or family history of any autoimmune or infectious disease, were included in the study. The response rate was about 85%. There was no statistically significant difference between the numbers of Males and Females in the patient and the control group. Higher age group of controls were, however, preferred to rule out their possibility of developing T1D at a later date after the collection of their blood samples as normal healthy controls. Since the control samples have been collected over a period of 5–6 years, all the controls who had the predisposing DRB1*0301 allele and were below the age of 30 years at the time of blood collection have been followed up till the date of analysis to make sure that they did not develop diabetes.

### Genotypic Analysis of *VDR* Polymorphisms

The genotypes for the four SNPs were determined by PCR amplification and restriction digestion of the PCR products with enzymes *FokI, BsmI, ApaI*, and *TaqI* as described earlier [23], [59] Briefly, 500 ng of DNA was amplified in 5 µl of 10X Thermo Pol reaction buffer supplemented with 2 mM MgSO_4_ (New England Biolabs), 5 pm of each primers, 0.25 mM of dNTPs, and 1.25 U of *Taq* DNA Polymerase (New England Biolabs), under standard conditions for 35 cycles in Perkin Elmer 2700 thermocycler. 1% agarose gel was run to confirm the amplification. To determine the presence of restriction site within an amplified product, a 5 µl aliquot of respective PCR product was digested with the respective restriction endonucleases. Figures 1A–D show the sizes of the restriction fragments obtained after digestion with the respective enzymes, the primers, their annealing temperatures for the PCRs and the temperatures at which restriction digestion was carried out. The interpretation of restriction fragments were done as shown in figures 1A–D.

### HLA-DRB1 Polymorphism

Alleles of *HLA-DRB1* locus were determined for 233 T1D patients and 191 controls. For 100 patient samples and 94 controls samples, the second exon of the *DRB1* gene was PCR amplified using standard conditions and hybridized with sequence specific oligonucleotide probes (SSOP) as described earlier [2], [60], [61]. The PCR products were run on 1% agarose gel to check for amplification. The amplified PCR products were dot blotted on Zeta probe membranes (Biorad), UV cross-linked (Syngene, USA) and hybridized with ^32^P- labeled probes for *DRB1*- generic, *DR2, DR4* and *DR52*- associated alleles.

For the remaining 133 patients and 97 control samples a Labtype SSO kit from One Lambda, (Canoga Park, CA, USA) was used according to the manufacturer's instructions as described earlier [2]. Briefly, 40 ng of DNA was amplified in master mix, primers and 2 µl of *Taq* DNA polymerase in Perkin Elmer 2700 thermal cycler. The amplified product was run on 1% agarose gel to confirm amplification. 5 µl of amplified product was hybridized with appropriate amount of multiplex beads conjugated with oligonucleotide probes, in hybridization buffer at 60°C for 15 minutes followed by washing and incubation with 50 µl 1X SAPE solution (Steptavidin Phycoerythin) at 60°C for 5 minutes. Fluorescence of the labeled beads was acquired on Luminex 2.2 flow cytometer (Luminex corporation, Austin, TX). Acquired data was analysed using Labtype software provided by One Lambda for analysis of *HLA* alleles. Some of these samples that were typed using PCR-SSOP earlier were re-typed using Luminex method to study the concordance using Luminex multiplex beads conjugated with oligonucleotide probes, since this was a new method being adopted in the lab to avoid using radioactivity. With 98% concordance between the two methods, Luminex method was used for the rest of the samples. A few samples were typed a second time using Luminex, for *HLA-DR* alleles, just to confirm the reproducibility.

### *HLA-DRB1* Promoter Sequencing

Sequencing of the *DRB1* promoter region was done to determine the VDRE variants in the North Indian population. The *HLA-DRB1* promoter region was amplified from 3 T1D subject samples and 3 control samples homozygous for HLA-DRB1*0301 using the primers described earlier, 5′-TTTCAGAAGAGGACCTT-3′ and 5′- CTTACGTCGGGTGTCCC-3′ [62]. Briefly, 100 ng of DNA was amplified in 2.5 µL of 10X standard Taq Buffer, supplemented with 3 mM MgCl_2_ (New England Biolabs), 2 pmoles of each primer, 0.25 mM dNTPs, and 1.25U of Taq DNA polymerase (New England Biolabs), under standard conditions at 54°C annealing, for 35 cycles in Eppendorf Mastercycler Gradient. Sequencing (using Sanger's di-deoxy chain termination method) of the PCR products was performed using primers internal to the amplification primers which were designed using NCBI primer blast. The sequences of the internal primers used are: 5′-ATACAGCATCTCTGACCAGC-3′, 5′-TCAGCACCATCAGTGTCA-3′. Sequences were aligned using ClustalW2, and the presence of a VDRE was confirmed in-silico using JASPAR_CORE version 3.0 database using default conditions [28].

### Expression Analysis Using Flow Cytometry and Real Time PCR

HLA-DRB1-*0301 homozygous B-lymphoblastoid cell line (B-LCL IHW09023) VAVY and DUCAF (B-LCL IHW09019) were cultured in RPMI 1640 with L-glutamine, supplemented with 15% fetal bovine serum and 100 nM sodium pyruvate at 37°C in humidified 5% CO_2_. Cells were incubated with Calcitriol (Sigma) at a final concentration of 100 nM for 24 hours from a 10 µM stock in ethanol. Control cells were treated with the same volume of absolute ethanol alone.

### Flow Cytometry

Flow cytometry was performed using anti-human HLA-DR antibody conjugated with PE (BD biosciences). 7×10^5^Cells were stained with anti-HLA-DR antibody as per manufacturer's recommendations. Briefly, the cells were washed with Dulbecco's Phosphate buffered saline (DPBS) twice and resuspended in 100 µl of staining buffer containing 0.5% BSA in DPBS. To this 10 µl of anti-DR antibody-PE was added and incubated on ice for 30 minutes. The cells were washed with 0.5% BSA in DPBS, fixed in 2% paraformaldehyde for 10 minutes on ice and acquired on a BD-LSR flow cytometer. Data analysis was performed on Win-MDI 2.9 software.

### Real-Time Analysis

Total RNA was isolated from cells using Trizol (Invitrogen) method. The RNA was purified using Qiagen RNeasy Mini column purification kit, and reverse transcribed using Superscript III First Strand Kit (Invitrogen). Real Time PCR analysis was performed on the ABI 7000 cycler (95°C 10 min Activation, 40 cycles: 95°C 15 secs Denaturation, 60°C 1 min Anneling/Extension), using Maxima SYBR Green qPCR Master Mix (Fermentas) with the following primers:

18s r RNA: Forward 5′ CGAAAGCATTTGCCAAGAAT 3′

Reverse 5′ AGTCGGCATCGTTTATGGTC 3′

HLA-DRB1: Forward 5′ TTAAGCTGCCACAAGAAACG 3′

Reverse 5′ TGTTCTCCAGCATGGTGTGT 3′

Expression level changes were calculated as fold-change in the level of transcript between treated and untreated samples and normalized to 18s rRNA levels. ΔCt values were obtained by subtracting the average Ct values of triplicate SYBR green assays for 18s rRNA from that of the corresponding HLA-DR. Fold change was calculated by using 2^−ΔΔCt^, where ΔΔCt represents ΔCt of treated - ΔCt of untreated control.

### Statistical Analysis

Chi-Square test or Fisher's exact test (whenever the numbers were five or less), were used to determine the significance of differences between the patient and control groups. p values were corrected using Boneferroni's correction by multiplying the p values with the number of alleles tested for the locus. Odds ratios were calculated using Woolf's method, with Haldane's modification wherever the numbers were five or less as described earlier [63]. Further, the Haplotype analysis was done using the SHEsis software freely available at (http://202.120.7.14/analysis/myAnalysis.php). [27] Hardy-Weinberg equilibrium was also calculated for both patients and controls using SHEsis software [27]. Additionally, Famhap (http://famhap.meb.uni-bonn.de) was used to confirm the frequencies of the haplotypes. Since both Famhap and SHEsis were giving similar results, we carried out the rest of the analysis using SHEsis only.

Interaction of two unlinked loci was tested using “LD based statistics” [25] using the following formulae
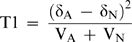



Where δ_A_ is the measure of LD between two unlinked loci a and b in cases and is calculated as
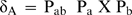


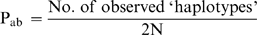



Where P_ab_ is the observed ‘haplotype’ frequency of alleles a and b at two unlinked loci (in this case *HLA-DR3* and the *VDR* haplotype/allele) and P_a_ is the gene frequency of the concerned allele at one locus and P_b_ is the gene frequency of the unlinked allele on the other locus. Here the word ‘haplotype’ does not necessarily mean that they are on the same chromosome as conventionally thought, rather simultaneously present alleles of two unlinked loci which may be associated with each other.

Similarly δ_N_ is the measure of LD between two unlinked loci a and b in controls.

V_A_ and V_N_ are the estimators of variance in patients and controls respectively and are calculated as:
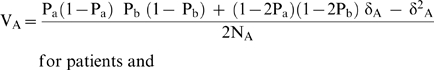


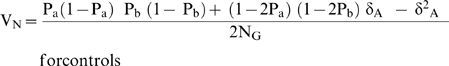



N_A_ and N_G_ are the number of samples tested in patients and control groups respectively.

The test Statistic T1 is asymptotically distributed as a central χ^2^_(1)_ distribution under the null hypotheses of no interaction between the two unlinked loci [25].
